# Editorial: Psychiatric Disorder in Veterans

**DOI:** 10.3389/fpsyt.2021.666719

**Published:** 2021-04-27

**Authors:** Giulio M. Pasinetti, Kyle J. Trageser, Joyce M. Harary, Theresa C. Gleason

**Affiliations:** ^1^Department of Neurology, Mount Sinai School of Medicine, New York, NY, United States; ^2^Geriatric Research, Education and Clinical Center, James J. Peters Veterans Affairs Medical Center, Bronx, NY, United States; ^3^Office of Research and Development, Department of Veterans Affairs, Washington, DC, United States

**Keywords:** veterans, mood disorders, PTSD, psychiatry, public policy, stress

## Introduction

Mental health issues in Veterans of the Armed Forces have wide-ranging effects. Factors related to military service, especially from combat related deployments, include environmental exposures, stressors, and physical traumas and injuries, with potential to places service members at heightened risks for the development of neuropsychiatric disorders. There exists a great need to identify contributory factors, elucidate mechanisms, investigate treatments, and apply these findings in a multiple tiered approach, aimed at translating basic research findings to evidence-based applications in the clinic. To improve mental health outcomes for Veterans, public health interventions and supportive public policies are vital to ensure access to the most effective mental health services. This special issue represents a collection of original research articles and review articles from esteemed colleagues who have conducted scientific research highly focused on the common goals of addressing, understanding and improving the mental health of Veterans.

## Prevalence of Mental Health Disorders in Veterans

Mental health illness presents many challenges for society, and is of imminent importance within the U.S. Veteran population. Our military service men and women are placed under unique conditional stressors that have put veterans at higher risk of developing neuropsychiatric illness including depression, anxiety, and posttraumatic stress disorder (PTSD) (1). Understanding the contributory factors leading to a predisposition for the development of neuropsychiatric illness in this population is a challenging but critical component in developing effective therapeutics and interventions to treat mental health among veterans.

The insecurity, trauma, and pressure of warfare causes tremendous stress, which makes it an important focus of research efforts. Following Israeli Veterans from the battlefield, to their reintegration into civilian life, Solomon provides a meaningful review of systematic trauma research focusing on the psychopathological effects on Israeli veterans. Her work is distinct, as Israel and its inhabitants have lived through periods of intense war, making the country a natural stress laboratory with a unique opportunity to study a war-like environment (2) and test potential prevention and treatment solutions.

## Contributing Factors

Various military service-related factors including stress, physical trauma, and environmental exposures have been investigated for their contributory roles in the development of mental health pathologies in Veterans. This nexus of predisposing factors is evident in the pathogenesis of Gulf War Illnesses (GWI). GWI is a chronic, multi-symptom disorder affecting approximately one third of all troops deployed to the Persian Gulf during the Gulf War (1990-91). Neuropsychiatric effects include memory dysfunction, PTSD, depression, and anxiety. The similarity of neuroinflammatory changes observed in both neuropsychiatric conditions and GWI is at the forefront of Trageser et al. review. As there is currently no approved treatment for GWI, these commonalities may be leveraged as a target for therapeutic interventions.

Veterans are also at a heightened risk of PTSD compared to the general population; Veterans deployed during Operation Enduring Freedom and Operation Iraqi Freedom exhibited a prevalence of 15.8%, compared to a 6.1% lifetime prevalence in the general population (2, 3). Toward investigating PTSD in veterans, Ypsilanti et al. examined the associations of self-disgust, loneliness, and mental health issues in Veterans with PTSD. In this study, the loneliness and anxiety symptoms of Veterans with PTSD is shown to be mediated by self-disgust measures. These findings have wide-ranging impacts for targeting treatments for the improvement of these symptoms.

Various neuropsychiatric disorders place individuals at risk for the development of Alzheimer's disease and other dementias (4). With the increased incidence of mental health issues in Veterans, in addition to other heightened risk factors, this link represents a vital area of research. Zhu and Sano describe these risk factors and approaches to prevent the development of Alzheimer's disease in the Veteran population.

## Moving Forward

To address the wide-reaching impacts of mental health disorders in the Veteran population, innovative, and evidence-based, treatment strategies and public policies should be employed. Alternative and innovative interventions interventions are essential in the treatment of psychiatric disorders in Veterans. Rodriguez et al. explores the utilization of a PTSD Service Dog intervention in Veterans afflicted with PTSD. Service dogs trained in a variety of therapy tasks were successfully able to attenuate many symptoms of PTSD, including those of hypervigilance and anxiety (5).

Toward identifying key factors that are integral for improving the mental health of Veterans, Fogle et al. has provided an invaluable review of all studies conducted by the National Health and Resilience in Veterans Study (NHRVS). To reduce the risks associated with mental disorders, it is essential to promote community integration and social engagement, and help individuals develop positive protective psychosocial characteristics.

Federal funding for research that is specific to improving mental health care for Veterans is essential. Activities such as collaborations across the major funders, the Department of Veterans Affairs (VA), Department of Defense and National Institutes of Health have been highly fruitful in identifying effective solutions. One of the major research programs, the Congressionally Directed Medical Research Program (CDMRP), is described by Lane et al. as highly collaborative and focused on making research awards tailored to areas of greatest need for active duty military and Veterans.

As described in Carroll et al. suicide prevention is the number one clinical priority of the VA. A multi-tiered approach aimed at addressing suicidality in Veterans both in the clinic and from community-based interventions is essential to prevent suicide in Veterans, as well as the general population. The VA's suicide prevention program has served as a model for others.

## Conclusion

Mental health issues in the Veteran population represent a significant social, economic, and public health burden, with wide ranging impacts. Research aimed at elucidating the mechanisms, the development of interventions, and application of public health initiatives and public policies targeting mental health issues in Veterans is vital. These articles featured by our colleagues represent novel strategies aimed toward achieving the goals of advancing evidence-based prevention and treatment solutions.

## Author Contributions

All authors listed have made a substantial, direct and intellectual contribution to the work, and approved it for publication.

## Conflict of Interest

The authors declare that the research was conducted in the absence of any commercial or financial relationships that could be construed as a potential conflict of interest.

## References

[1] Seal KH Metzler TJ Gima KS Berenthal D Maguen S Marmar CR. Trends and risk factors for mental health diagnoses among iraq and afghanistan veterans using department of veterans affairs health care, 2002–2008. Am J Public Health. (2009) 99:1651–8. 10.2105/AJPH.2008.150284 19608954 PMC2724454

[2] Dursa EK Reinhard MJ Barth SK Schneiderman AI. Prevalence of a positive screen for PTSD among OEF/OIF and OEF.OIF-Era veterans in a large population-based cohort. J Trauma Stress. (2014) 27:542–9. 10.1002/jts.21956 25267288

[3] Goldstein RB Smith SM Chou SP Saha TD Jung J Zhang H. The epidemiology of DSM-5 posttraumatic stress disorder in the United States: results from the National Epidemiologic Survey on Alcohol and Related Conditions. Soc Psychiatry Psychiatr Epidemiol. (2016) 51:1137–48. 10.1007/s00127-016-1208-5 27106853 PMC4980174

[4] Bennett S Thomas AJ. Depression and dementia: cause, consequence or coincidence? Maturitas. (2014) 79:184–90. 10.1016/j.maturitas.2014.05.009 24931304

[5] Rodriguez KE LaFollette MR Hediger K Ogata N O'haire ME. Defining the PTSD service dog intervention: perceived importance, usage, and symptom specificity of psychiatric service dogs for military veterans. Front Psychiatry. (2020) 11:1638. 10.3389/fpsyg.2020.01638 32849004 PMC7396623

